# Cyclodextrins as Multifunctional Platforms in Drug Delivery and Beyond: Structural Features, Functional Applications, and Future Trends

**DOI:** 10.3390/molecules30143044

**Published:** 2025-07-20

**Authors:** Iuliana Spiridon, Narcis Anghel

**Affiliations:** ”Petru Poni” Institute of Macromolecular Chemistry, Grigore Ghica-Voda No. 41A, 700487 Iasi, Romania; spiridon@icmpp.ro

**Keywords:** inclusion complexes, stimuli-responsive systems, nanocarriers

## Abstract

Cyclodextrins (CDs) are cyclic oligosaccharides capable of forming inclusion complexes with various guest molecules, enhancing solubility, stability, and bioavailability. This review outlines the structural features of native CDs and their chemically modified derivatives, emphasizing the influence of functionalization on host–guest interactions. Synthetic approaches for CD derivatization are summarized, with attention to recent developments in stimuli-responsive systems and targeted drug delivery. Analytical techniques commonly employed for characterizing CD complexes, such as spectroscopy, thermal analysis, and molecular modeling, are briefly reviewed. Applications in pharmaceutical formulations are discussed, including inclusion complexes, CD-based conjugates, and nanocarriers designed for solubility enhancement, controlled release, and site-specific delivery. Special consideration is given to emerging multifunctional platforms with biomedical relevance. The regulatory status of CDs is addressed, with reference to FDA- and EMA-approved formulations. Safety profiles and toxicological considerations associated with chemically modified CDs, particularly for parenteral use, are highlighted. This review presents an integrative perspective on the design, characterization, and application of CD-based systems, with a focus on translational potential and current challenges in pharmaceutical development.

## 1. Introduction

Cyclodextrins (CDs) are cyclic oligosaccharides first identified in the late 19th century by Antoine Villiers through the enzymatic degradation of starch [1]. Initially regarded as mere curiosities, detailed structural and chemical analyses later revealed their significant potential, particularly through the mid-20th century contributions of Schardinger, who thoroughly characterized and defined these compounds [2]. CDs represent a unique class of naturally occurring molecules derived primarily from starch via enzymatic actions involving cyclodextrin glycosyltransferases (CGTases). CGTases facilitate the cleavage and subsequent cyclization of starch-derived linear glucan chains, thus forming cyclic structures [3,4,5,6].

Structurally, CDs are composed of α-(1,4)-linked D-glucopyranose units forming cyclic molecules with a distinct toroidal geometry. This shape imparts a conical form with a hydrophilic exterior and a relatively hydrophobic central cavity [7,8,9,10]. Such structural configurations bestow CDs with extraordinary molecular encapsulation properties, where the hydrophobic internal cavity selectively accommodates a wide range of guest molecules via non-covalent interactions [11,12,13]. This unique ability to encapsulate diverse molecules supports their extensive applications across multiple scientific domains, notably pharmaceutical sciences, food technology, agrochemical formulations, environmental remediation, and analytical chemistry [14,15,16,17,18,19,20,21].

**Figure 1:**
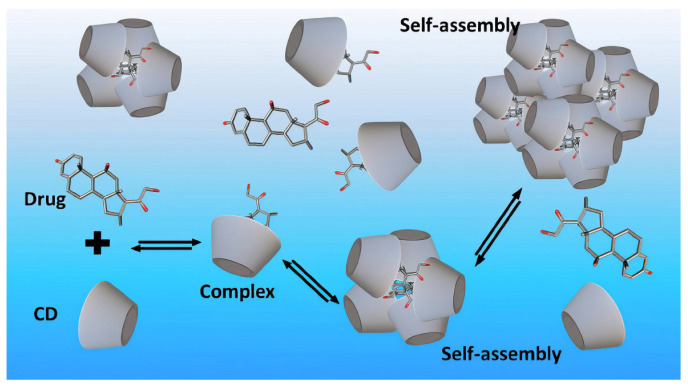
Formation of a cyclodextrin inclusion complex in an aqueous solution and self-assembly of cyclodextrin complexes. Reproduced from Ref. [21], under the terms of the Creative Commons Attribution License (CC BY).

Commonly encountered CDs are α-, β-, and γ-cyclodextrins, comprising six, seven, and eight glucopyranose units, respectively [22,23]. Among these, β-cyclodextrin (β-CD) has attracted considerable attention due to its favorable cavity size, superior guest-binding versatility, and robust structural stability, making it particularly suitable for pharmaceutical [24,25] and industrial applications [26] (Figure 1). However, native β-CD’s moderate aqueous solubility (approximately 18.5 g/L at room temperature) imposes limitations on its broader utility, necessitating chemical modifications that enhance solubility, stability, and selectivity [26,27,28]. These chemically modified derivatives, such as hydroxypropyl-β-CD (HP-β-CD, >600 g/L), sulfobutylether-β-CD (SBE-β-CD, >500 g/L), and randomly methylated-β-CD (RM-β-CD, 500–700 g/L), offer significantly improved solubility profiles and therapeutic efficacy, expanding the practical usage of CDs substantially [29,30,31,32].

In supramolecular chemistry, CDs occupy a pivotal role due to their ability to establish stable inclusion complexes through non-covalent interactions (van der Waals forces, hydrophobic effects, hydrogen bonding, and electrostatic interactions) [33,34,35,36]. Such host/guest complexes profoundly alter the physicochemical characteristics of encapsulated molecules, including enhanced solubility, bioavailability, chemical stability, and reduced volatility, toxicity, and undesired side effects. Consequently, CDs have emerged as indispensable agents within drug delivery systems, significantly advancing therapeutic outcomes through innovative formulation strategies such as enhanced drug stability, controlled release, targeted delivery, and improved patient compliance [37,38,39,40].

Recent advancements underline the multifunctionality of CDs beyond traditional pharmaceutical contexts, extending into novel applications in nanomedicine, diagnostics, bioimaging, smart material engineering, and stimuli-responsive drug release systems [41,42,43,44]. CDs have been effectively integrated into nanoscale drug carriers, acting as building blocks for sophisticated supramolecular architectures, including polymeric micelles, nanogels, nanoparticles, and liposomes, thereby facilitating targeted delivery and precision medicine approaches [45,46,47]. Their inherent biocompatibility and biodegradability further reinforce CDs’ suitability for biomedical applications, leading to increasing interest in their use for drug delivery.

Moreover, CDs play a significant role in diagnostic applications by enhancing the sensitivity, specificity, and accuracy of analytical assays through their ability to encapsulate and stabilize fluorescent and chromophoric probes, thereby improving detection limits and assay performance [48,49,50,51]. In smart material engineering, CDs contribute to the design of environmentally responsive systems capable of modulating drug release in response to external stimuli such as pH, temperature, enzymatic activity, and light irradiation, offering versatile and precise therapeutic interventions [52,53,54,55].

Future trends in CD research indicate a strong trajectory towards multifunctional and stimuli-responsive systems, encompassing advanced biomedical applications, environmental remediation technologies, agricultural enhancements, and sustainable industrial processes [7,17,56,57]. Notably, the exploration of hybrid and conjugated CD-based materials, incorporating nanotechnology and polymer science principles, is expected to unlock unprecedented applications, especially in the fields of personalized medicine and environmental sustainability [58,59,60]. Such advancements require an in-depth understanding of CD structure/function relationships, synthesis methodologies, and derivatization strategies to optimize their performance and application-specific properties.

Additionally, addressing existing limitations such as host/guest specificity, controlled encapsulation/release dynamics, and regulatory considerations will be crucial for future development. Advanced computational modeling and simulation techniques, coupled with high-throughput synthesis and screening methodologies, offer promising approaches to overcoming these challenges, enabling the design of CDs with tailored functionalities and enhanced performance [61,62,63].

Cyclodextrins have become multifunctional materials applicable across diverse fields. Their derivatization strategies and tunable structural features, covered in detail in Section 2.4 and Section 3, support a broad spectrum of drug delivery solutions.

The ongoing evolution in CD chemistry and application fields promises sustained growth and expansion, advancing new therapeutic frameworks, sustainable environmental approaches, and transformative technologies.

Table 1 provides a concise overview of the main application areas of cyclodextrins across pharmaceutical, industrial, environmental, and consumer sectors.

This review distinguishes itself from previous cyclodextrin literature by offering a comprehensive and integrative perspective that spans structural characterization, derivatization strategies, and the latest developments in stimuli-responsive and targeted delivery systems. Unlike prior reviews that tend to focus on specific application domains (e.g., pharmaceuticals, food, or environmental uses) or singular aspects of cyclodextrin chemistry, our review systematically consolidates emerging advances across diverse sectors, while emphasizing recent innovations such as multi-stimuli responsive systems, supramolecular engineering, and nanomedicine integration. We also discuss analytical methodologies in greater detail, correlating structure/function relationships with host/guest dynamics, and offer forward-looking insights into hybrid systems and regulatory challenges. This integrative approach aims to serve as both a foundational and forward-guiding resource for researchers and practitioners working with cyclodextrins in multidisciplinary contexts.

## 2. Structural Features and Types of Cyclodextrins

### 2.1. Chemical Structure and Conformation

Enzymatically derived from starch, cyclodextrins are formed through the action of cyclodextrin glucanotransferase (CGTase), an enzyme that cleaves linear glucans and reassembles them into toroidal, ring-like structures composed of D-glucopyranose units linked via α-1,4-glycosidic bonds [24,77]. The three naturally occurring CDs, α-CD, β-CD, and γ-CD, contain six, seven, and eight glucopyranose units, respectively.

**Figure 2:**
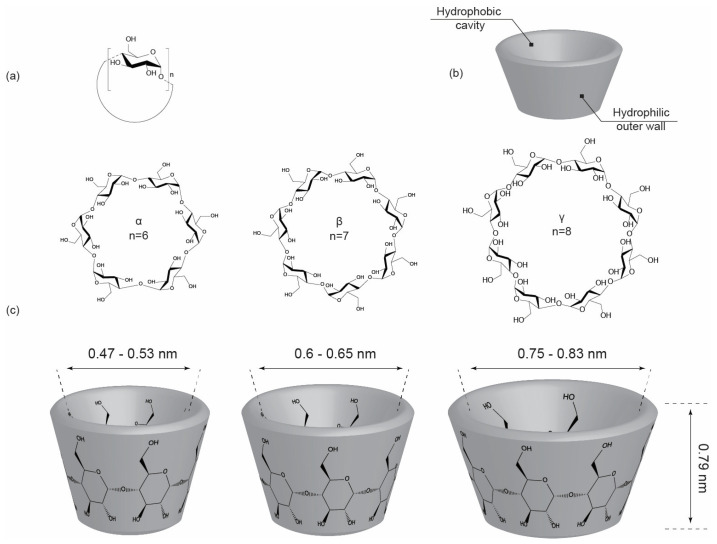
Representations of: (**a**) the general chemical structure; (**b**) the tridimensional structure of cyclodextrins; (**c**) chemical structure and dimensions for α-, β-, and γ-cyclodextrin (n = 6, 7, and 8, respectively).

Each glucopyranose unit adopts a 4C_1_ chair conformation, and the overall CD structure resembles a truncated cone. The primary hydroxyl groups (C_6_–OH) are located on the narrow rim, while the secondary hydroxyl groups (C_2_–OH and C_3_–OH) occupy the wider rim, conferring asymmetry and amphiphilicity to the molecule [78,79]. These hydroxyl groups are involved in intra- and intermolecular hydrogen bonding, leading to a relatively rigid framework, although CDs retain some conformational flexibility that may influence their binding interactions (Figure 2).

X-ray crystallography and nuclear magnetic resonance (NMR) studies have confirmed that the conformation of CDs is slightly distorted from perfect symmetry due to these hydrogen bonds and the repulsion between adjacent hydroxyl groups [77,80,81]. The internal cavity diameter ranges from 4.7 to 5.3 Å in α-CD, 6.0 to 6.5 Å in β-CD, and 7.5 to 8.3 Å in γ-CD, while the height is approximately 7.9 Å for all three. These cavity dimensions determine the suitability of each CD for encapsulating guest molecules of different sizes and shapes [82,83].

**Figure 3:**
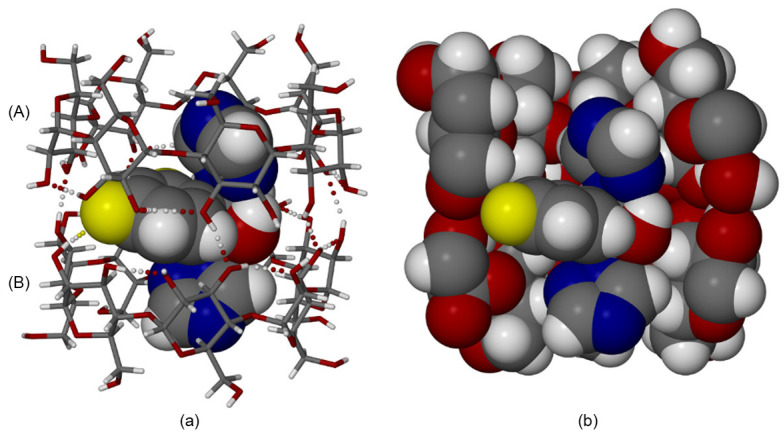
The dimeric complex TBCDFLU: (**a**) Stick representation of the two independent host molecules (A and B), with the guest molecule FLU shown in space-filling mode; (**b**) Cutaway view from the same angle, displaying both host and guest molecules in space-filling representation. Reproduced from Ref. [84], under the terms of the Creative Commons Attribution License (CC BY).

**Figure 4:**
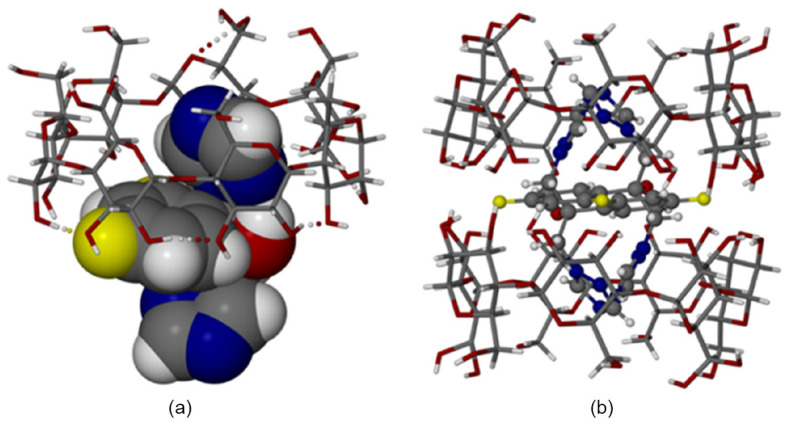
The complex MBCDFLU: (**a**) The asymmetric unit, with water oxygen atoms omitted for clarity; (**b**) The (β-CD)_2_–FLU complex unit, showing the disordered components of the FLU guest molecule. Reproduced from Ref. [84], under the terms of the Creative Commons Attribution License (CC BY).

**Figure 5:**
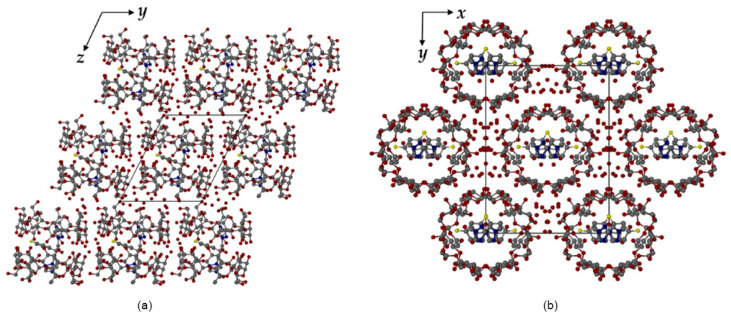
Crystal packing arrangements: (**a**) TBCDFLU viewed along the (100) projection; (**b**) MBCDFLU viewed along the (001) projection. Hydrogen atoms are omitted for clarity. Water oxygen atoms are shown as red spheres. The C-centered arrangement of the complex units is clearly visible in (**b**). Reproduced from Ref. [84], under the terms of the Creative Commons Attribution License (CC BY).

To illustrate the structural characteristics and crystallographic behavior of cyclodextrin–fluconazole inclusion complexes, Figure 3, Figure 4 and Figure 5 are reproduced from the original study [84]. These figures provide detailed representations of the host–guest interactions, asymmetric units, and crystal packing arrangements observed in both TBCDFLU (tert-butylated β-cyclodextrin–fluconazole complex) and MBCDFLU (methylated β-cyclodextrin–fluconazole complex) systems. Together, they offer valuable insights into the molecular architecture, disorder phenomena, and packing symmetry that influence the physicochemical properties of cyclodextrin-based formulations.

Understanding the precise conformation of CDs is crucial for applications in supramolecular chemistry, drug delivery, catalysis, and sensing, where the host/guest interaction dynamics depend strongly on spatial compatibility and conformational stability.

### 2.2. Inner Hydrophobic Cavity and Outer Hydrophilic Surface

The molecular architecture of cyclodextrins presents a unique amphiphilic profile that is crucial to their function as host molecules. The inner cavity, formed by the axial orientation of hydrogen atoms and ether-like glycosidic oxygen bridges, is relatively nonpolar and hydrophobic, mimicking a microenvironment similar to that of nonpolar solvents. This hydrophobic cavity can accommodate a variety of lipophilic guest molecules, from small aromatic compounds and aliphatic chains to volatile oils, drugs, and dyes. This inclusion ability represents the basis of CDs’ utility in pharmaceutical, food, and industrial formulations [14,24,72,85].

Surrounding this cavity, the outer surface of the CD molecule is densely populated with hydroxyl groups, the primary hydroxyls on the narrower rim and secondary hydroxyls on the wider rim. These hydroxyls confer hydrophilicity and water solubility, especially for α- and γ-CD. The combination of a hydrophobic core and a hydrophilic exterior enables CDs to bridge polar and nonpolar environments, acting as molecular carriers in aqueous media [86,87,88,89].

The spatial arrangement of these polar and nonpolar regions is not merely passive. Molecular modeling and thermodynamic studies have demonstrated that upon inclusion of a guest molecule, the water molecules occupying the CD cavity are displaced. These cavity-bound water molecules are relatively high in enthalpy and entropy due to the constraints imposed by the narrow, apolar cavity. Their release into bulk solvent during inclusion complexation contributes significantly to the negative Gibbs free energy change (ΔG), making the process thermodynamically favorable [90,91,92,93].

**Figure 6:**
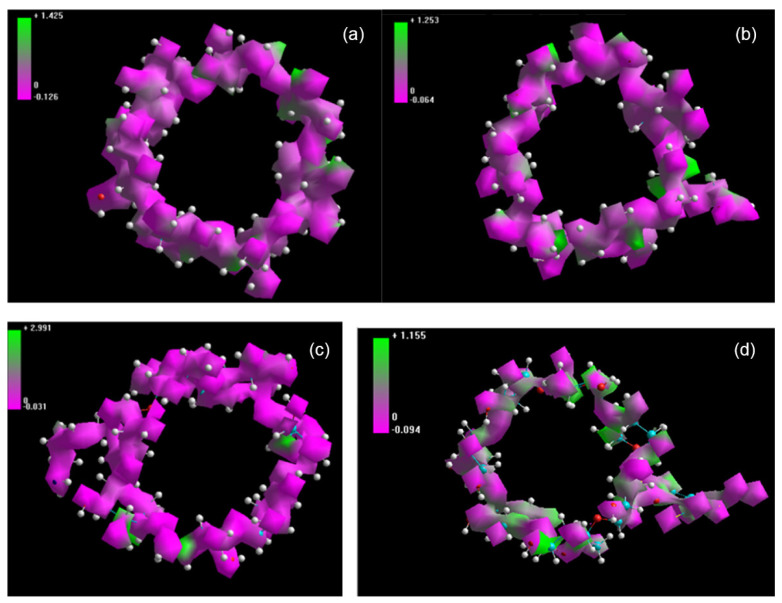
Molecular electrostatic surface potential maps of (**a**) β-CD, (**b**) CM-β-CD, (**c**) MDE-β-CD (mono(2,6-di-O-methyl)-β-cyclodextrin), and (**d**) DFB-β-CD (difluorobenzyl-β-cyclodextrin). Pink regions indicate areas of high electron density (electron-rich), while green regions represent electron-deficient zones. The accompanying scale bar denotes the range of electrostatic potential values at each surface position. Reproduced with permission from Ref. [94]. Copyright 2025, Elsevier.

Figure 6 presents molecular electrostatic surface potential maps of native β-cyclodextrin and selected derivatives, highlighting the distribution of electron-rich and electron-deficient regions that underlie their amphiphilic behavior [94].

Furthermore, the size and shape of the cavity, coupled with the position and orientation of the hydroxyl groups, dictate the host’s selectivity toward different guests. For instance, β-CD is most suitable for molecules like ibuprofen, curcumin, or benzoic acid, while γ-CD is more accommodating to larger biomolecules such as peptides or macrolides. The dynamics of inclusion are influenced by structural compatibility, as well as the presence of hydrogen bond donors or acceptors on the guest molecule that can interact with rim hydroxyls [95,96,97].

Advanced spectroscopic methods such as 2D-ROESY NMR, circular dichroism, and isothermal titration calorimetry (ITC) have been employed to study these interactions in detail. These methods provide insight into the stoichiometry, orientation, binding affinity, and energetics of complexation. Importantly, CDs do not chemically alter the guest but rather form non-covalent, reversible complexes that can protect labile molecules from hydrolysis, oxidation, and photodegradation.

In recent years, nanotechnology has leveraged this amphiphilic structure in the creation of supramolecular assemblies such as micelles, nanoparticles, and hydrogels. CDs serve not only as solubilizers but also as building blocks in responsive materials that change behavior under stimuli such as pH, temperature, or ionic strength. The interplay between hydrophilic surfaces and hydrophobic cavities enables stimuli-responsive release systems that are currently under investigation for targeted drug delivery and biosensing applications.

### 2.3. Solubility and Inclusion Complexes

The aqueous solubility of native cyclodextrins varies dramatically and is a key determinant of their application profile. γ-CD, with its large cavity and flexible structure, is the most soluble of the native CDs (~23 g/100 mL at 25 °C), followed by α-CD (~14 g/100 mL), while β-CD is the least soluble (~1.85 g/100 mL). The low solubility of β-CD is attributed to strong hydrogen bonding in the crystal lattice and a more rigid, less hydrated conformation [98,99,100]. These differences in solubility affect the selection of a particular CD for formulation purposes.

Inclusion complex formation between CDs and guest molecules leads to changes in both the solubility and stability of the guest. Through the encapsulation of hydrophobic regions within the cavity, CDs enhance the apparent solubility of otherwise poorly soluble compounds. This not only improves bioavailability but also protects the guest from environmental degradation, light-induced decomposition, and enzymatic hydrolysis [101,102].

Table 2 summarizes the solubility enhancement profiles of various cyclodextrin types, highlighting their relative efficiency in improving the aqueous solubility of guest compounds.

The thermodynamics of inclusion is governed by the release of structured water molecules from the CD cavity and the entropic gain from increased disorder in the system. Enthalpic contributions arise from van der Waals interactions, hydrophobic effects, and potential hydrogen bonding at the CD rims. Inclusion complex formation is typically a spontaneous process (negative ΔG), and the stability constants (K_s_) range from 10^2^ to 10^5^ M^−1^ depending on the guest, CD type, and solution conditions [14,106,107,108].

Stoichiometries are most commonly 1:1, but 1:2 and 2:1 complexes are also documented, especially in the case of guests with multiple aromatic rings or elongated geometry [21,109,110,111,112]. Characterization of these complexes is achieved through a combination of methods, including phase solubility analysis, differential scanning calorimetry (DSC), powder X-ray diffraction (PXRD), and spectroscopic techniques like UV-Vis, FTIR, and NMR.

Beyond pharmaceuticals, inclusion complexes are used extensively in the food industry to stabilize flavors and aromas, in cosmetics to encapsulate volatile oils, and in textiles to control the release of active agents. In environmental science, CDs form complexes with organic pollutants, facilitating their extraction, separation, or degradation.

Advanced systems in which CDs are grafted onto polymers or embedded in nanosponges to form three-dimensional networks that retain inclusion capacity while offering greater mechanical and chemical stability have also been developed. These systems are particularly promising for sustained and controlled drug delivery, where the release of the guest is governed by diffusion, degradation, or environmental triggers such as pH or temperature.

### 2.4. Natural vs. Chemically Modified Cyclodextrins

While native CDs have desirable inclusion properties, their use is constrained by several limitations, including low solubility (notably for β-CD), non-specificity in binding, and variable safety profiles at higher doses. Chemically modified CDs (CM-CDs) have been developed to overcome these limitations by introducing functional groups that alter their physicochemical and pharmacokinetic properties [24,66,73,113,114].

Hydroxypropyl-β-cyclodextrin (HP-β-CD) is one of the most important derivatives, prepared by reacting β-CD with propylene oxide. The hydroxypropyl groups increase aqueous solubility to over 50 g/100 mL and reduce crystallinity, enabling better complexation and easier formulation in aqueous systems. HP-β-CD is approved for parenteral, oral, and ocular use, with applications in solubilizing poorly soluble drugs such as dexamethasone and itraconazole [115,116].

Methylated-β-CD (M-β-CD) features methylation at the hydroxyl positions. Depending on the degree of substitution, methylation improves solubility and inclusion efficiency. However, complete methylation can lead to cytotoxicity and membrane lysis, especially in parenteral applications. Randomly methylated CDs (RM-β-CD) are used primarily in analytical chemistry, especially in chiral separation techniques such as capillary electrophoresis [117].

Sulfobutyl ether-β-CD (SBE-β-CD) introduces negatively charged sulfonate groups, significantly enhancing solubility and adding electrostatic interaction capabilities. SBE-β-CD has high biocompatibility and is used in several FDA-approved injectable products, such as voriconazole. The anionic nature of SBE-β-CD also improves complexation with cationic drugs [118,119,120,121,122].

**Figure 7:**
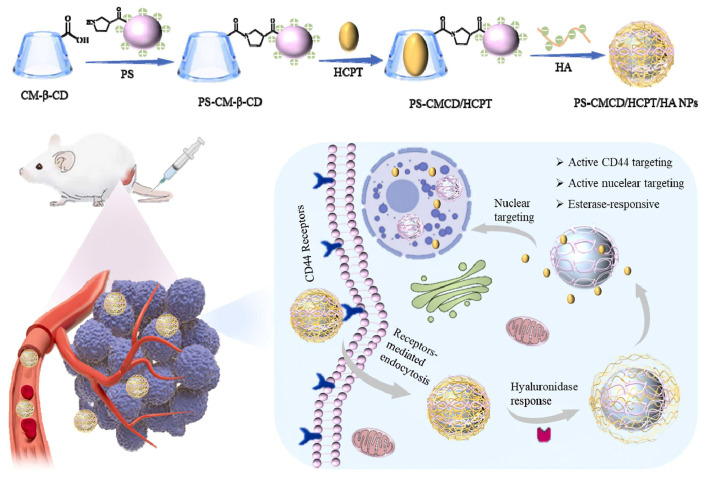
Diagrammatic representation of preparation and tumor therapy of PS-CMCD (protamine-modified carboxymethyl-β-cyclodextrin)/HCPT (hydroxycamptothecin)/HA (hyaluronic acid) NPs (nanoparticles). Reproduced with permission from Ref. [124]. Copyright 2025, Elsevier.

Other functionalized CDs include carboxymethyl-, acetyl-, amino-, and polymer-bound derivatives, each with specific benefits (Figure 7). For example, carboxymethyl-β-CD is advantageous for pH-responsive release [123], while polymeric CD-based nanocarriers enable targeted delivery by attaching ligands or antibodies [124,125,126,127].

These derivatives expand the utility of CDs into highly specialized areas such as gene delivery (by forming complexes with nucleic acids), enzyme immobilization, diagnostic imaging, and responsive materials for smart therapeutics. Moreover, combinatorial libraries of CD derivatives are being explored through high-throughput screening to tailor host/guest systems with optimized performance for emerging challenges in precision medicine and environmental remediation.

Importantly, each modified CD must be rigorously evaluated for toxicity, metabolism, and excretion pathways. Regulatory acceptance varies by country and application route, and safety profiles are contingent on substitution patterns, degrees of substitution, and residual reagents from synthesis.

In conclusion, chemically modified CDs offer unparalleled versatility, allowing the design of molecular systems tailored for solubilization, delivery, sensing, or separation, far beyond the capabilities of natural CDs alone.

## 3. Synthesis and Derivatization

Cyclodextrins acquire functional versatility through diverse derivatization routes, which are explored in detail throughout Section 3. This enables tailoring their physicochemical properties for specific pharmaceutical or materials science applications.

This section outlines the key methods for CD synthesis, strategies for chemical modification, and recent advances in developing stimuli-responsive (“smart”) CD derivatives [128,129,130].

### 3.1. Enzymatic Production via Cyclodextrin Glucanotransferase (CGTase)

The most common and industrially scalable method for producing natural CDs involves the enzymatic conversion of starch by cyclodextrin glucanotransferase (CGTase), an enzyme that belongs to the glycoside hydrolase family 13 (α-amylase family). CGTase catalyzes intramolecular transglycosylation, cleaving α-1,4 glycosidic bonds in linear starch chains and facilitating the cyclization reaction that forms CDs, particularly α-CD (6 glucose units), β-CD (7 glucose units), and γ-CD (8 glucose units) [131,132].

This enzymatic process comprises multiple activities, including hydrolysis, cyclization, coupling, and disproportionation. Cyclization is key to CD formation and is influenced by multiple factors, including the source of the CGTase enzyme, substrate concentration, pH, temperature, and the presence of complexing agents [133,134]. CGTases from *Bacillus macerans*, *Bacillus circulans*, and *Thermoanaerobacterium thermosulfurigenes* are well-characterized and exhibit varying specificities for different CD ring sizes [135,136,137].

Optimizing the enzymatic environment, such as through the use of organic solvents, salting-out agents, or complexing molecules, can steer the reaction towards the preferred CD size. For instance, toluene or methyl orange can favor β-CD production by selectively precipitating or stabilizing the desired product. Industrial-scale purification often employs membrane filtration, ethanol precipitation, and column chromatography to isolate high-purity CDs [138,139].

Recombinant DNA technology has enhanced CGTase production and thermostability. Immobilized enzyme systems and continuous-flow bioreactors are under investigation to reduce production costs and increase yields. Such advances are crucial for sustainable, scalable CD manufacturing, especially in pharmaceutical-grade applications [140].

### 3.2. Strategies for Chemical Modification

Native CDs offer inclusion potential but face limitations such as poor aqueous solubility and rigid cavity structures (see Section 2.3 for initial discussion).

**Figure 8:**
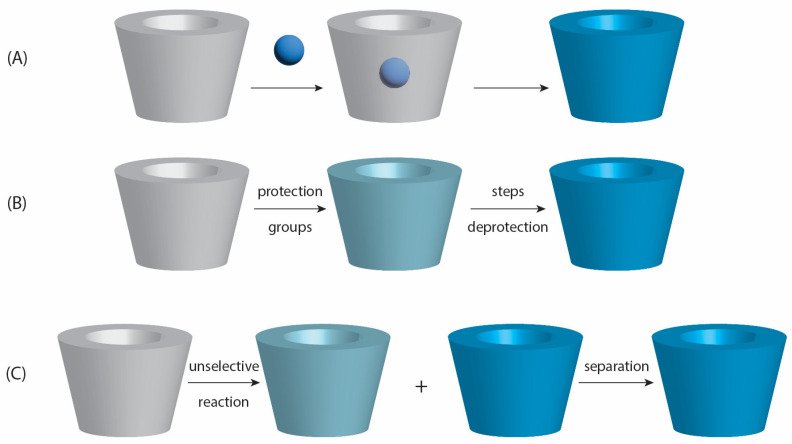
Strategies for cyclodextrin modification: (**A**) ‘clever’ for methods using inclusion of reagents to obtain selectivity; (**B**) ‘long’ for methods using protection groups; (**C**) ‘sledgehammer’ for methods requiring extensive separation of isomers. Color scheme: gray—native, unmodified cyclodextrin; intermediate teal—cyclodextrin undergoing functionalization, protection, or partial derivatization; cyan—final modified or fully functionalized cyclodextrin.

Chemical modification of CDs allows fine-tuning of their properties by introducing functional groups onto the hydroxyl moieties located at the C_2_, C_3_ (secondary face), and C_6_ (primary face) positions of the glucose units [129,141,142] (Figure 8).

Hydroxyalkylation is a widely employed method involving the reaction of CDs with epoxides such as propylene oxide or ethylene oxide. This process yields hydroxypropyl- or hydroxyethyl-CDs, significantly enhancing solubility and reducing crystallinity. Hydroxypropyl-β-cyclodextrin (HP-β-CD) is especially prominent due to its high aqueous solubility (>500 mg/mL) and minimal toxicity, making it suitable for oral, ocular, and parenteral drug delivery [143,144,145,146,147].

Methylation can be performed using dimethyl sulfate or methyl iodide under basic conditions, yielding randomly methylated (RM-β-CD) or per-methylated derivatives. Methylation disrupts hydrogen bonding and alters hydrophobicity, enhancing binding affinity for lipophilic drugs. However, full methylation can lead to cytotoxicity and is thus limited to specific uses like chiral separation in analytical chemistry [29,148,149,150,151].

Table 3 presents a summary of reaction yields and key physicochemical parameters associated with commonly used cyclodextrin (CD) derivatization pathways, as reported in the literature.

Additional derivatization techniques include carboxymethylation, introducing pH-sensitive carboxylic groups, which improve solubility in basic conditions and enable ionic interactions; sulfobutylation, achieved via reaction with 1,4-butanesultone, yielding sulfobutyl ether-β-CD (SBE-β-CD), an anionic derivative with excellent solubilizing power and low toxicity used in FDA-approved injectable drugs (e.g., voriconazole) [66,121,156,157,158]; acetylation, which reduces aqueous solubility, enabling CD use in hydrophobic environments or for delayed-release systems; amination, which allows CD conjugation with peptides, antibodies, or other targeting ligands, facilitating targeted drug delivery; and polymer grafting, enabling CDs to be embedded in nanosponges, dendrimers, or nanoparticles, enhancing multivalency and structural integrity [159,160,161,162,163,164].

The degree of substitution (DS) and positional selectivity are critical for defining the behavior of CD derivatives. Characterization is commonly performed using NMR spectroscopy, MALDI-TOF mass spectrometry, and elemental analysis. Chromatographic techniques to determine molecular weight distribution and substitution pattern are also used [142,165,166].

### 3.3. CDs Stimuli-Responsive Derivatives

Stimuli-responsive or “smart” cyclodextrins represent a new frontier in host/guest chemistry, where CDs are engineered to alter their behavior in response to external stimuli such as pH, temperature, light, redox potential, or enzymatic activity. These responsive systems offer precise spatial and temporal control over guest molecule encapsulation and release, with promising implications for targeted drug delivery, diagnostics, biosensing, and adaptive materials [41,54,167,168].

pH-responsive CDs are functionalized with groups such as carboxylic acids or tertiary amines, which ionize at specific pH values. For example, carboxymethylated CDs can exhibit pH-dependent complexation behavior, ideal for releasing drugs in the acidic microenvironments of tumors or inflamed tissues [169,170].

Temperature-responsive CDs are often linked to polymers like poly(N-isopropylacrylamide) (PNIPAM), which exhibit a lower critical solution temperature (LCST). Below the LCST, the system is hydrated and swollen; above it, the polymer collapses and can trigger guest release. These systems are suitable for pulsatile drug delivery and thermoresponsive gels [171,172].

Light-responsive CDs are functionalized with photochromic groups like azobenzene or coumarin. Upon light irradiation, these groups undergo reversible isomerization, altering their binding to CDs and allowing optical control over guest molecule capture and release [173,174,175,176,177].

**Figure 9:**
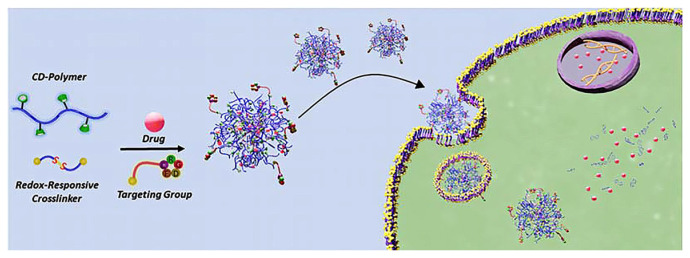
Illustrative depiction of the stepwise construction of drug-loaded nanogels with targeting capability. Reproduced with permission from Ref. [181]. Copyright 2025, Elsevier.

Redox-responsive CDs are engineered with disulfide bridges or ferrocene moieties. In the presence of reductive agents like glutathione, commonly elevated in intracellular environments, disulfide bonds cleave, releasing the guest. This strategy is widely employed in cancer nanomedicine [178,179,180,181,182,183] (Figure 9).

Enzyme-responsive CDs are conjugated to substrates specific to overexpressed enzymes (e.g., proteases, glycosidases), triggering release in pathological conditions. For instance, CD/peptide conjugates cleaved by matrix metalloproteinases (MMPs) enable tumor-selective delivery [60,62,184,185].

Multi-responsive systems, combining, for example, pH and redox sensitivity, allow for even finer control and adaptability in complex biological environments. These systems have been integrated into supramolecular hydrogels, vesicles, micelles, and layer-by-layer assemblies for diverse biomedical and material science applications [53,186,187,188].

In addition to therapeutic delivery, smart CDs are being explored in biosensors, self-healing materials, smart textiles, and stimuli-adaptive coatings. Their modularity and biocompatibility help to position them as key building blocks in the next generation of smart materials.

Despite significant advances in the synthesis and functionalization of cyclodextrins, the scale-up of CD-based systems for industrial or clinical use remains challenging. Major obstacles include batch-to-batch variability, complex purification of chemically modified derivatives, and low yields in multi-step reactions. For instance, the production of stimuli-responsive CD derivatives often involves labor-intensive protection/deprotection steps and costly reagents, which limit scalability [53,186,189]. Moreover, the control of substitution degree and site-selectivity during functionalization (e.g., for sulfobutyl or methylated CDs) is difficult to reproduce at large scale [66,121,190,191,192], affecting consistency and regulatory approval. In nanocarrier systems, reproducibility of nanoparticle size, encapsulation efficiency, and drug release profiles also pose challenges in GMP-compliant manufacturing [193,194,195,196]. These issues underline the need for scalable, cost-effective, and environmentally sustainable synthetic protocols for industrial translation of CD-based technologies [197,198,199,200].

## 4. Analytical Techniques for Cyclodextrin Characterization

Comprehensive characterization of cyclodextrins (CDs) and their inclusion complexes is essential for understanding their physicochemical behavior, structural modifications, and host/guest interaction mechanisms. A broad range of analytical techniques is employed to elucidate the structure, substitution pattern, thermodynamics, and complexation properties of native and modified CDs. This section presents key methods used for CD analysis and their relevance in studying inclusion phenomena, offering a framework for rational design and optimization of CD-based systems.

### 4.1. Characterization Techniques

Nuclear Magnetic Resonance (NMR) spectroscopy remains a cornerstone for cyclodextrin characterization. In ^1^H-NMR, the chemical environment of CD protons is sensitive to inclusion events, with H-3 and H-5 protons, located inside the cavity, exhibiting notable upfield shifts upon guest encapsulation. ^13^C-NMR provides further structural insight, especially when assessing chemically modified CDs, by revealing chemical shift variations at C2, C3, and C6 positions. Two-dimensional NMR techniques such as ROESY (Rotating-frame Overhauser Effect Spectroscopy) and NOESY (Nuclear Overhauser Effect Spectroscopy) are powerful tools to establish proximity correlations, thus determining the geometry, depth, and orientation of the guest within the CD cavity [201,202,203,204].

**Figure 10:**
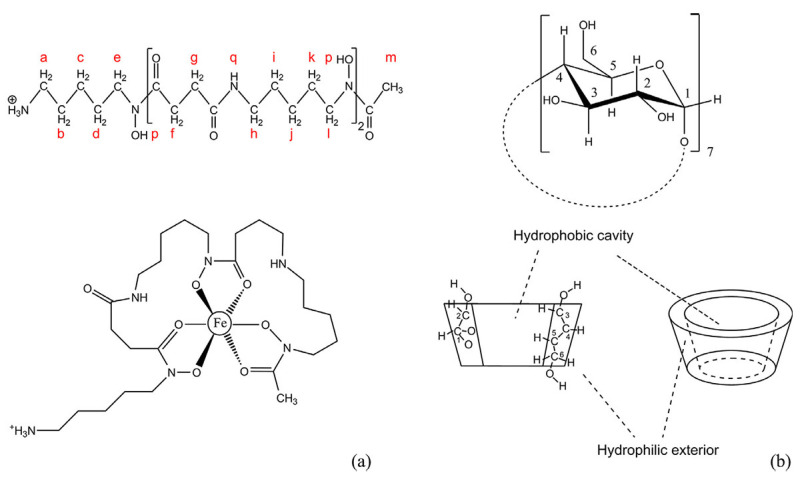
(**a**) Chemical structure of protonated desferrioxamine B and its iron(III) complex, ferrioxamine B. (**b**) Structure of β-cyclodextrin, highlighting one α-D-glucopyranose subunit and its toroidal conformation. The wider and narrower openings of the torus expose secondary and primary hydroxyl groups to the solvent, respectively. Proton positions relevant to the NMR study are also indicated. Reproduced with permission from Ref. [203]. Copyright 2025, Elsevier.

**Figure 11:**
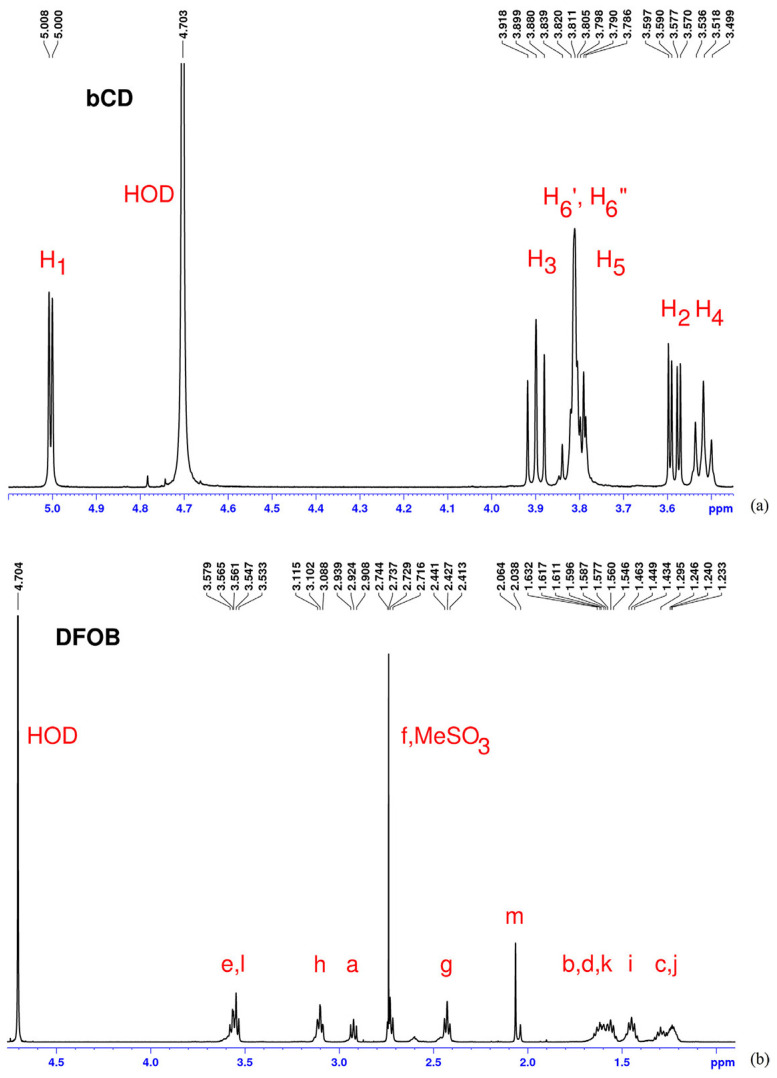
500 MHz ^1^H NMR spectra of pure (**a**) β-cyclodextrin (β-CD) and (**b**) desferrioxamine B (DFOB) recorded in D_2_O. Reproduced with permission from Ref. [203]. Copyright 2025, Elsevier.

To support the inclusion complex formation between desferrioxamine B (DFOB) and β-cyclodextrin (β-CD), Figure 10 and Figure 11 illustrate the chemical structures involved and the ^1^H NMR spectra of pure components, respectively [203]. Figure 10 highlights the molecular features relevant to the NMR study, while Figure 11 presents the spectral evidence confirming interaction between the host and guest molecules in aqueous solution.

Fourier Transform Infrared Spectroscopy (FTIR) is extensively used for confirming complexation and functional group substitution. Inclusions are evidenced by shifts in key vibrational bands, such as carbonyl stretches in guest molecules and the broad OH stretching of CDs. FTIR also aids in detecting the presence of specific linkages in chemically modified CDs, like ether, ester, or sulfonate groups [205,206].

Mass Spectrometry (MS), particularly matrix-assisted laser desorption/ionization time-of-flight (MALDI-TOF) and electrospray ionization (ESI-MS), is indispensable for determining molecular weights and identifying substitution degrees. MS can also distinguish between free and complexed forms of a guest molecule and assess the uniformity of CD derivatives [207,208,209,210].

Differential Scanning Calorimetry (DSC) offers thermal fingerprints of inclusion complexes. A marked shift or disappearance of the melting point of the guest in the DSC thermogram, along with potential shifts in CD thermal transitions, supports successful encapsulation. Combined with Thermogravimetric Analysis (TGA), DSC allows for moisture content and thermal degradation analysis [211,212,213].

Powder X-ray Diffraction (XRD) characterizes the crystalline state of CDs and their complexes. Inclusion often disrupts the host’s crystal lattice, producing amorphous or differently ordered structures. The appearance or disappearance of diffraction peaks signals structural rearrangements upon complexation [214,215,216].

Isothermal Titration Calorimetry (ITC) provides a direct, label-free measurement of the thermodynamics of host/guest interactions. ITC captures enthalpy (*ΔH*), entropy (*ΔS*), binding constant (*K*_a_), and stoichiometry in a single titration experiment under physiological conditions. It is especially valuable in drug design, where precise thermodynamic data are required [217,218,219].

Other techniques include UV-Vis and fluorescence spectroscopy for monitoring spectral shifts, fluorescence quenching or enhancement upon complexation; Circular Dichroism (CD) for assessing changes in chiral environments and complexation with optically active guests; Raman spectroscopy, as a complementary vibrational technique to FTIR, especially for aqueous samples; and Dynamic Light Scattering (DLS), for particle size analysis when CDs are incorporated into nanoscale carriers.

The combined application of these analytical tools ensures multidimensional validation of CD complexes, supporting both fundamental research and regulatory-compliant product development.

### 4.2. Host/Guest Interaction Studies

Understanding the nature and strength of host/guest interactions in CD systems is crucial for tailoring their application in solubilization, stabilization, drug release, and sensing. These interactions are typically non-covalent and reversible, involving hydrophobic effects, van der Waals forces, hydrogen bonding, and dipole/dipole interactions.

The 2D NMR (ROESY/NOESY) provides critical information about spatial proximity between guest protons and internal protons of the CD (H-3 and H-5), offering qualitative and semi-quantitative data on insertion depth, guest orientation, and symmetry of binding. Coupled with molecular modeling, these experiments enable visualization of host/guest complexes [220,221].

FTIR and Raman spectroscopy serve as spectroscopic fingerprints to identify interaction-induced vibrational shifts. When combined with chemometric analysis (e.g., PCA or PLS), they offer robust tools for discriminating complexed vs. free forms.

UV-Vis and fluorescence spectroscopy are frequently used in titration experiments to calculate binding constants and monitor real-time complexation. Complex formation may cause bathochromic/hypsochromic shifts, intensity changes, or new absorption bands. In fluorescence-based assays, guest inclusion can result in either quenching or enhancement, depending on the environment [222,223,224,225].

ITC reveals the thermodynamic landscape of complexation, indicating whether the interaction is enthalpy-driven (typically for hydrogen bonding or polar guests) or entropy-driven (often for hydrophobic effects). This aids in the rational design of CD-based formulations for controlled release or targeting [38,226].

Surface Plasmon Resonance (SPR) and Quartz Crystal Microbalance (QCM) are increasingly used for real-time monitoring of binding kinetics, especially in sensor applications.

Advanced host/guest studies now incorporate molecular dynamics simulations, docking studies, and machine learning algorithms to predict binding affinities and complex stability, further enhancing the design of efficient CD carriers.

### 4.3. Stoichiometry and Binding Constants

Accurate knowledge of the stoichiometry and binding constants of CD complexes is essential for designing effective formulations, ensuring therapeutic efficacy, and predicting release kinetics. While 1:1 complexes are most common due to size compatibility, 1:2 (guest/host—one CD molecule encapsulates two guest molecules) and 2:1 (host/guest—two CD molecules encapsulate a single guest) systems are observed with elongated or symmetrical guests, or in polymeric and multivalent systems.

The stoichiometry (how many host and guest molecules are involved) depends on several key factors, with molecular size and shape of the guest being the most influential (Table 4).

Phase solubility studies, pioneered by Higuchi and Connors, remain the gold standard for determining stoichiometry and calculating the apparent stability constant (*K*_s_).

**Figure 12:**
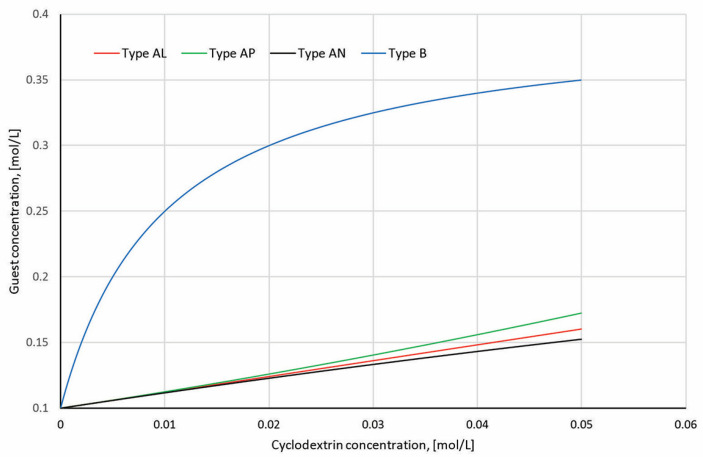
Phase solubility diagram illustrating the effect of cyclodextrin concentration on guest solubility, showing distinct profiles for complexation types AL, AP, AN, and B.

Here is a comparative plot of phase solubility diagrams: type AL—linear increase—typical 1:1 soluble complex; type AP—positive deviation—possible higher-order or cooperative binding; type AN—negative deviation—reduced solubility at higher cyclodextrin concentrations; type B—plateau—limited solubility due to complex precipitation or insoluble complex formation (Figure 12).

A-type phase solubility diagrams indicate soluble complexes, while B-type diagrams reveal limited solubility. The slope of the linear portion of an AL diagram is used to calculate *K*_s_ via the equation (Equation (1)):

(1) $$K_{s}=\frac{slope}{S_{0}\times \left(1-slope\right)}$$

where *S*_0_ is the solubility of the free guest [11,12].

Job’s method (continuous variation) is another classical approach for determining binding ratios, especially when combined with spectroscopic techniques.

Benesi–Hildebrand analysis applies to UV-Vis and fluorescence titrations for 1:1 systems, providing a linear method for *K_s_* estimation.

ITC simultaneously provides *K_s_*, *ΔH*, *ΔS*, and stoichiometry (n), offering a comprehensive thermodynamic profile. High binding constants (>10^4^ M^−1^) are desirable for stabilization, but may require triggers (e.g., pH or enzyme) for controlled release.

Accurate determination of these parameters not only informs formulation strategy but also influences regulatory approval, particularly for injectable or complex CD-drug systems.

Emerging techniques, such as high-throughput microcalorimetry, affinity capillary electrophoresis (ACE), and NMR titration with chemometric deconvolution, allow for parallel screening of multiple complexes, accelerating formulation development.

## 5. Applications in Drug Delivery

Cyclodextrins have emerged as versatile and powerful excipients in pharmaceutical sciences due to their unique capability to form inclusion complexes with a wide variety of poorly water-soluble drugs. This ability is rooted in their hydrophobic internal cavity and hydrophilic external surface, which enable the entrapment of non-polar guest molecules, improving their aqueous solubility, stability, and bioavailability. Their application extends across multiple delivery routes, including oral, parenteral, topical, ophthalmic, nasal, pulmonary, and transdermal administration. CDs can enhance drug targeting, modulate drug release kinetics, reduce irritation, mask unpleasant tastes and odors, and even contribute to the stabilization of biologics. As a result, they are increasingly integrated into advanced drug delivery systems, ranging from conventional dosage forms to highly engineered nanosystems for targeted medicine.

Furthermore, CDs offer a platform for the integration of multi-functional characteristics, combining solubilization, stabilization, and targeting within a single delivery vehicle. The incorporation of CDs into nanocarriers, micelles, vesicles, and lipid-based systems offers a significant step in achieving improved therapeutic outcomes and patient compliance. Their generally recognized as safe (GRAS) status and extensive regulatory acceptance further position them as ideal pharmaceutical excipients for future drug delivery innovations.

### 5.1. Solubility Enhancement

One of the major pharmaceutical applications of CDs is to improve the solubility of poorly water-soluble drugs, which represent more than 40% of new chemical entities. CDs form non-covalent inclusion complexes by hosting drug molecules within their hydrophobic cavity, thereby enhancing apparent solubility and dissolution rates. This phenomenon has been extensively used for drugs such as itraconazole, carbamazepine, nifedipine, and valsartan, where the resulting complexes facilitate better absorption and bioavailability [235,236,237,238].

Native CDs like α-, β-, and γ-CDs offer basic inclusion capabilities, but their solubility limitations have led to the development of various chemically modified derivatives such as hydroxypropyl-β-cyclodextrin, sulfobutyl ether-β-cyclodextrin, and randomly methylated β-cyclodextrin, which exhibit superior aqueous solubility and inclusion efficiency. These derivatives allow for the formation of more stable and concentrated solutions, improving both the pharmacokinetics and the ease of formulation.

Ternary complexes, incorporating a CD, drug, and water-soluble polymer (e.g., HPMC or PVP), have also shown promise in significantly enhancing solubility and maintaining supersaturation. These systems have enabled the successful formulation of solid dispersions and orally disintegrating tablets for molecules with extremely poor aqueous solubility.

In recent years, amorphous solid dispersions stabilized by CDs have gained traction for improving drug dissolution profiles. Spray-drying and hot-melt extrusion techniques are commonly employed to prepare such systems, enabling scalable production of solubility-enhanced pharmaceuticals. Moreover, the use of CDs in nanosuspension formulations resulted in particle size reduction and surface modification, further accelerating dissolution rates and absorption.

The drug solubility in aqueous solutions is an important parameter of medicines. The development of CD inclusion complexes offers a simple method to enhance the administration of insoluble drugs. Song et al. [239] have developed new bright NIR-II-emitting gold nanoclusters (Au NCs) protected by cyclodextrin. The material surface exhibited good stability and efficient renal clearance of Au and also allowed high-affinity protein labeling via host/guest complexation without modification treatments. This material demonstrated a high-efficiency NIR-II imaging to track in vivo physiological behavior of the proteins after injection, as well as a good NIR-II visualization of the targeted tumor.

A recent paper reported methyl-β-CD as a promising excipient to be applied in inhalable cannabinoid formulations [240]. Methyl-β-CD and cannabidiol formed a 1:1 inclusion complex that should be stored at < 70% humidity. In spite of this limitation, it exhibited good aerosol performance. Other authors reported that the solubility of curcumin was improved by complexation with hydroxypropyl-β-CDs [241]. As a result of the increase in curcumin solubility, the permeation of curcumin from the curcumin/CD inclusion complex was significantly higher across the olfactory epithelial tissue, as well as the respiratory epithelial tissue, as compared to that of curcumin. The inclusion complex of 2,6 di-O-methyl-β-CD with abiraterone acetate [242] resulted in the enhancement of its solubility. This was reflected in the abiraterone acetate in vitro release. Thus, its complexation with CD-derivative augmented the drug dissolution rate, with promising potential in practical application in prostate cancer treatment. The solubility and in vitro release rates of oxymetholone were improved by its inclusion in the carboxymethyl-β-CD cavity [243]. The hydrophobic interactions, electrostatic interactions, as well as hydrogen bonding between the drug and carboxymethyl-β-CD improved encapsulation efficiency. Additionally, a prolonged in vitro release profile was evidenced, making the oxymetholone–carboxymethyl-β-CD inclusion complex a promising formulation to improve the therapeutic efficacy of oxymetholone.

The inclusion complexes of CDs and insulin demonstrated that CDs enhanced the stability, solubility, and bioavailability of insulin formulations [244]. Additionally, these formulations can be administered via different channels (orally and via the mucous membranes), thus improving the effectiveness of insulin delivery.

Ibrutinib, a drug used in B-cell malignancies, is less commonly used due to low oral bioavailability [245]. It seems that its incorporation in hydroxypropyl-β-CD using 1,1′-carbonyldiimidazole as a cross-linking agent resulted in the controlled drug release at the targeted site. Hydroxypropyl-β-CD improves the drug’s aqueous solubility while the cross-linked network produces nano-channels in the polymer mesh structure. Thus, it is possible to reduce the frequency of the doses administered, as well as to increase patient compliance.

The inclusion complex of CDs and Pluronic127 and α-CD/β-CD [246] sustained acyclovir delivery, representing an interesting approach to develop materials with topical applications [247].

Albendazole showed remarkable activity against hepatocellular carcinoma and colorectal cancer cells, but presents poor water solubility. It was found [248] that its inclusion in methyl-β-CD improved albendazole’s water solubility by 150,000 times as compared to albendazole alone, making this complex a potential anti-anthelmintic and anti-tumor agent.

Genistein with amino-appended β-CDs [249] presented an aqueous solubility 1000 times higher compared to genistein alone, while the complexes are safe on the human normal cell line in terms of cytotoxicity. Other genistein/CD formulations [250] exhibited higher in vitro antioxidant and cell viability activity against the MCF-7 breast cancer cell line than genistein.

### 5.2. Stabilization of Labile Drugs

CDs offer protective encapsulation for drugs that are prone to hydrolysis, oxidation, photodegradation, or thermal degradation. The inclusion complex acts as a molecular shield, reducing the drug’s exposure to environmental aggressors. For example, ascorbic acid, prone to oxidative and photolytic degradation, has shown increased stability when complexed with HP-β-CD [251].

In parenteral formulations, CDs can stabilize peptides and proteins by reducing aggregation, enzymatic degradation, and denaturation during storage and administration. For instance, insulin formulations have been stabilized using CDs, improving their shelf life and pharmacological performance [252]. The stabilization effect is often synergistically improved by combining CDs with cryoprotectants and antioxidants during lyophilization.

CDs also protect drugs against pH-induced degradation. Omeprazole, a proton pump inhibitor unstable in acidic conditions, maintains its activity when encapsulated in a CD complex. Similarly, prostaglandins, which are chemically unstable and highly sensitive to light and heat, have shown increased shelf life when formulated with CDs.

Furthermore, CDs enhance the chemical and enzymatic stability of nucleic acid therapeutics such as siRNAs, antisense oligonucleotides, and plasmid DNA. Inclusion complexation reduces susceptibility to nucleases and allows for more consistent delivery performance in gene therapy protocols. Such stabilization is critical in ensuring successful transfection and gene silencing effects.

Emerging studies have also shown that CDs can inhibit crystallization and polymorphic transitions of drug molecules, thereby stabilizing their amorphous forms in solid-state formulations. This property significantly contributes to maintaining consistent bioavailability over shelf life.

CDs have also been explored in combination with antioxidants, chelating agents, and preservatives to create multi-mechanistic stabilization systems for complex formulations. These hybrid systems are particularly beneficial for ophthalmic, injectable, and dermal products that demand high stability and prolonged shelf life.

Table 5 summarizes experimental data on improvements in drug formulation parameters, including bioavailability, half-life extension, and solubility enhancement.

Beyond stabilization of actives, CDs can also protect excipients and formulation adjuvants, improving the overall integrity of pharmaceutical products. Their inclusion into vaccine formulations, including mRNA-based vaccines, has proven to stabilize fragile nucleic acid constructs during thermal stress and storage.

Complexation with hydroxylpropyl-β-CD and sulfobutylether-β-CD resulted in the improvement stability of a 3-hydroxy-2-pyridinaldoxime hybrid [259] in plasma while allowing its passage across the blood/brain barrier. This approach became a promising solution for organophosphorus compound (usually present in pesticides) poisoning.

Sulfobutylether cyclodextrin as a complexing agent was also able to enhance the solubility and bioavailability of azithromycin [260]. NMR spectra evidenced the occurrence of a conformational modification involving the carbonyl region of the macrocycle, which is protected from hydrolytic attack.

The complex of methyl-β-cyclodextrin with oseltamivir [261] improved drug encapsulation efficiency, as well as its biological activity.

Both β-CD and carboxymethyl-β-CD were able to encapsulate ethinyl estradiol. The release process was controlled by diffusion and erosion processes, but the drug was slowly released from the β-CD inclusion complex, while it was released faster from the other one, probably due to the steric hindrance of CM-β-CD and the more porous structure of β-CD [262].

Another study [243] confirmed a three times higher solubility of oxymetholone in carboxymethyl-β-CD/oxymetholone as compared to the β-CD/oxymetholone complex. The steroid nucleus of oxymetholone fitted well into the cavities of β-CD and carboxymethyl-β-CD, but the carboxymethyl-β-CD/oxymetholone complex presented higher aqueous solubility. According to phase solubility, the carboxymethyl-β-CD/oxymetholone complex exhibited slower oxymetholone release as compared to the β-CD/oxymetholone complex.

The 7-ethyl-10-hydroxycamptothecin presents a high tumor growth inhibition ability, but poor solubility and stability. Yang et al. [263] reported that CDs and their derivatives could help to design delivery systems that enable the clinical benefits of the drug.

Water-soluble anionic β-CDs derivative complexes, including voriconazole, miconazole, itraconazole, and amphotericin B, were evaluated in terms of solubility, cytotoxicity, and antifungal efficacy [264]. The aqueous solubility of the antifungal drugs was improved due to the hydrophobic and electrostatic interactions. The citric acid crosslinked β-CD including miconazole exhibited good drug loading and mucoadhesive properties, with a sustained release profile. The cytotoxicity and cytocompatibility studies demonstrated its safety while being effective against yeast and filamentous fungi.

### 5.3. Controlled Release Systems

**Figure 13:**
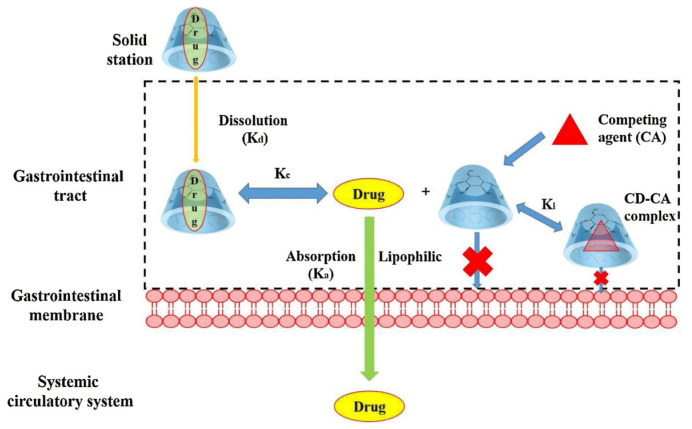
The process of the non-covalent system to release the anticancer drug. K_d_ is the dissolution rate constant; K_c_ is the stability constant of the complex of the drug with the CD; K_i_ is the stability constant of the complex of the competing agent with CD; K_a_ is the absorption rate constant. Reproduced with permission from Ref. [266]. Copyright 2025, Elsevier.

CDs are widely applied in the development of controlled release systems, enabling precise temporal drug administration. CDs can be incorporated into various delivery matrices such as hydrogels, nanosponges, microspheres, films, and implants. Their inclusion properties and ability to interact with polymers make them ideal candidates for designing formulations with delayed, extended, or pulsatile release profiles [265,266,267] (Figure 13).

Cross-linked CD nanosponges are among the most promising CD-based controlled release systems. These porous, three-dimensional polymeric networks trap drug molecules and release them over a prolonged period. Studies have shown their application in the sustained delivery of doxorubicin, tamoxifen, and dexamethasone [162].

CD/hydrogel composites have been used in the development of smart release systems that are responsive to pH, temperature, or glucose. For instance, CD–insulin complexes in pH-sensitive hydrogels have demonstrated on-demand insulin release under hyperglycemic conditions, representing a potential breakthrough for diabetes management [268].

Additionally, multilayer CD-based nanoparticles allow for the sequential release of multiple drugs. These systems are particularly advantageous in cancer therapy, where combinations of chemotherapeutic agents and immunomodulators need to be released at different times and cellular locations [269].

In oral delivery, CD-based tablets designed for colon targeting can bypass gastric degradation and release the drug in response to colonic pH and microbiota. These technologies have proven effective for drugs like budesonide and mesalamine used in inflammatory bowel disease [270].

Other approaches include the use of CD-grafted polymers to create depot formulations that provide zero-order release over weeks or months. Implantable CD-based depots have been tested for hormone therapies and pain management, enhancing compliance and reducing administration frequency.

Advanced CD delivery platforms have incorporated responsive release features such as redox-sensitive, enzyme-sensitive, and light-triggered mechanisms. These innovations enable on-demand release tailored to the pathophysiological environment, such as tumor-specific enzymes or oxidative stress markers.

Recently, cyclodextrin-based supramolecular hydrogels have gained attention due to their reversible, shear-thinning properties, which allow for injectable depot formation and in situ gelation [188]. These platforms are being explored for prolonged local delivery of antibiotics and anticancer agents.

The release of drugs is very important to establish optimal therapeutic treatment in order to ensure that drugs are delivered at the appropriate rate and maintain effective concentrations within the target area. In the next pages, information related to the capacity of CDs to facilitate controlled drug release through several mechanisms will be offered. It is worth mentioning that a major challenge is the restricted drug loading capacity of CDs due to their cavity dimension, which limits the quantity and size of guest drug molecules. This aspect has an important impact on the therapeutic dosages. Forming inclusion complexes of cyclodextrins with drugs that exhibit different dissociation rates or the use of stimuli-responsive moieties could release the drug under the action of specific triggers (temperature, changes in pH, the presence of specific enzymes).

**Figure 14:**
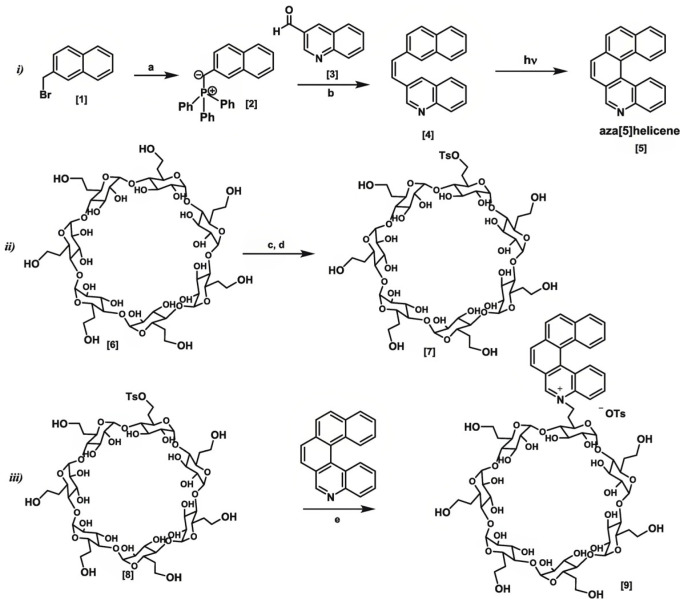
Synthesis of β-CD-Aza[5]helicene. Reagents and conditions: (**i**) synthesis of aza[5]helicene core—(a) triphenylphosphine, toluene, r.t, 24 h, yield 78%; (b) NaOMe, 3-quinolinecarboxaldehyde, CH3OH, refluxed 3 h, yield 88%; (**ii**) functionalization and macrocyclization of the helicene—(c) NaOH, p-TsCI, 3 h r.t; (d) HCl, 4 °C, overnight, yield 85%; (**iii**) host–guest complexation; (e) DMF, 90°, 24 h, yield 80%. Reproduced from Ref. [271], under the terms of the Creative Commons Attribution License (CC BY).

The ability of CDs to form inclusion complexes with drug molecules and the photoluminescence properties of Aza[5]helicene were combined to obtain a theranostic platform for gemcitabine, a metabolic nucleoside inhibitor used in cancer treatment [271]. It was found that the β-CD-Aza[5]helicene conjugate increased the therapeutic value of the drug, while its systemic toxicity was reduced (Figure 14).

Nanofibers obtained after grafting β-CD onto chitosan were efficient in the sustained release of indomethacin as an anti-inflammatory agent. The indomethacin solubility was improved while it was also protected against degradative processes. The materials have potential applications in wound healing, as well as in topical applications. A slow and sustained release of the drug from β-CD-grafted chitosan nanofibers into PBS (saline phosphate buffer) solution was recorded. The authors explained the improvement in the drug release process by the entangling of the drug inside the nanofibers and the long time necessary to migrate from inside the nanofiber to its surface [272]. Fenofibric acid, a lipid-lowering drug, was complexed with β-CD and its methyl derivative [273]. The drug release rate was influenced by the interaction mechanism between the host and guest. Thus, when the drug was included in β-CD, fenofibric acid was encapsulated within the interior cavity, resulting in a slower-releasing complex as compared with that formed when the drug was included in methyl β-CD. The drug incorporation into the CD cavity also enhanced the pharmacological profile of fenofibric acid.

Sun et al. [274] developed sialic acid-targeted cyclodextrin-based nanoparticles that were able to efficiently deliver colony-stimulating factor-1 receptor to M2 tumor-associated macrophages. M2 macrophages were reprogrammed to the immunostimulatory M1 phenotype through the downregulation of colony-stimulating factor-1 expression, thus enhancing the level of apoptosis in the prostate cancer cells.

Sulfonated CDs [275] were efficient against some viruses (herpes simplex virus, respiratory syncytial virus, dengue virus, and Zika virus). Their antiviral effect can be explained by the relatively short seven-carbon sulfonated alkyl chain reducing the number of interactions between the CD and virus, as well as by the linker, which, independent of the binding affinity, is a key element for the virucidal mode of action. The Janssen vaccine against SARS-CoV-2 infection (ad26.cov2.s), comprising 2-hydroxypropyl-β-cyclodextrin [276], re-opened a huge interest in CDs. Almeida et al. [277] stipulated that the inclusion complex comprising CDs and antiviral drugs protects drugs against degradation, and increases their solubility, bioavailability, and biological activity. At the same time, the use of CDs enlarges administration possibilities, thus avoiding side effects or problems associated with direct systemic administration.

### 5.4. Targeted Delivery Strategies

Functionalized CDs are being developed for site-specific drug delivery by conjugating them with ligands like folic acid, transferrin, RGD (short sequences of amino acids, specifically arginine-glycine-aspartic acid) peptides, and monoclonal antibodies. These constructs exploit receptor-mediated endocytosis to achieve targeted uptake by cancer cells, inflamed tissues, or brain endothelium [184,278,279,280].

For example, folate-conjugated HP-β-CD nanocarriers have been used for the targeted delivery of paclitaxel to folate receptor-overexpressing tumor cells. The inclusion complex protects the drug from systemic degradation and releases it selectively in the tumor microenvironment, thereby reducing off-target toxicity.

In neuropharmacology, CDs modified with glutathione or lactoferrin can cross the blood/brain barrier (BBB) and deliver neuroprotective agents such as curcumin or resveratrol to the central nervous system. This opens avenues for treating neurological diseases like Alzheimer’s and Parkinson’s.

Additionally, CDs are used in theranostic applications, where they act as both delivery carriers and imaging agents. CD complexes co-loaded with magnetic resonance contrast agents or fluorescent dyes allow simultaneous imaging and therapy, a concept increasingly used in personalized medicine.

Targeting inflamed tissues is another promising application. CDs functionalized with leukocyte-adhesion ligands can deliver anti-inflammatory drugs to arthritic joints or inflamed bowel regions. This compound has shown promising results in preclinical models of rheumatoid arthritis and Crohn’s disease.

Transdermal and mucosal delivery systems based on CDs have also been developed for localized action. These systems improve drug penetration across epithelial barriers while reducing systemic absorption. Such approaches have been beneficial in delivering corticosteroids and antifungals.

New-generation targeting systems include CDs conjugated with cell-penetrating peptides, aptamers, and CRISPR-Cas9 components, broadening the scope of genomic medicine and intracellular delivery. In cancer immunotherapy, CDs have been used to co-deliver antigens and adjuvants to dendritic cells, triggering robust T cell activation.

Recently, efforts have focused on CD-decorated liposomes, dendrimers, and solid lipid nanoparticles for dual or triple targeting, increasing both specificity and drug accumulation at pathological sites. These platforms integrate CD-mediated solubilization with ligand-guided delivery for optimized performance.

For effective diagnosis and treatment, drug delivery systems must deliver therapeutic agents to the desired target area in order to maximize the therapeutic efficacy while minimizing the side effects. The presence of numerous hydroxyl groups offers cyclodextrins multiple functionalization routes with targeting moieties, such as antibodies or peptides, capable of recognizing and binding to specific receptors or antigens on the surface of target cells, inflamed tissues, or specific cellular receptors.

Ou et al. [281] found that CDs can directly target tumor-promoting molecules like Galectin-3. This protein facilitates tumor progression by promoting cancer cell adhesion, migration, and invasion, leading to metastasis. The authors reported that after binding of CDs to the protein, its concentration in the tumor microenvironment is reduced, and cancer cells’ ability to produce metastasis is thus inhibited. A new β-cyclodextrin-aza[5]helicene conjugate as a platform for gemcitabine delivery was synthesized [271]. The photoluminescent properties of the aza[5]helicene, as well as the improved solubility and stability resulting from complexation within the β-cyclodextrin, resulted in an increased pharmacokinetic effect of gemcitabine, while its toxicity was reduced. Additionally, trackable drug delivery into cancer cells is possible due to the photoluminescence properties of aza[5]helicene. A new dry powder formulation consisting of paclitaxel, γ-cyclodextrin, and KOH [282] was developed for the treatment of pulmonary diseases. The authors demonstrated that paclitaxel presented improved pharmacokinetics and reduced the recurrence and deterioration of tumors in this formulation due to the enhanced solubility, as well as contact with the blood vessels on the lung surface over a large area. These characteristics resulted from paclitaxel encapsulation with a γ-CD metal/organic framework. The reprogramming of M2-like tumor-associated macrophages within the tumor microenvironment is a new trend in cancer therapy [283]. Paclitaxel was embedded into the inclusion complex consisting of arginine and biotin-modified hydroxypropyl-β-CD as the host, with benzimidazole and mannose-modified hyaluronic acid (BM-HA-Man) as the plug. It seems that BM-HA-Man could control the paclitaxel release in a pH-sensitive manner, and this ensures the release of the drug preferentially in the tumor microenvironment, inducing tumor cell apoptosis. As a result, the therapeutic efficacy is increased while the side effects of the treatment are reduced.

Sialic acid was conjugated with stearic acid-PEG-COOH and co-formulated with an amphiphilic cationic cyclodextrin to obtain nanoparticles for the delivery of CSF-1R siRNA [274]. The obtained nanoparticles exhibited the ability to enhance apoptosis in prostate cancer.

Table 6 summarizes representative examples of cyclodextrin/guest inclusion complexes relevant to drug delivery strategies described in this section, highlighting the type of cyclodextrin used and the functional benefits observed.

β-CD was modified with a peptide that mimics Glu-urea-Lys as a prostate-specific membrane antigen ligand. Fluorescein was caged into the functionalized β-cyclodextrin to evaluate its capability to deliver the dye selectively to the LNCap prostate cancer cells. As compared to non-tumoral Hek293T cells, the experiments evidenced an enhanced cytotoxicity of doxorubicin in LNCap cells [301]. Additionally, the functionalized β-CD allows doxorubicin to accelerate the induction of cell death due to the uptake of the antineoplastic drug by prostate cancer cells.

β-CD was used to connect a glycodendrimer with a ZnO core, and the obtained material was loaded with doxorubicin and methotrexate. It was evidenced that the π-π stacking interactions among both the drugs and the aromatic network of the carrier became weaker in the acidic pH of the cancer cells, thus favoring enhanced drug release, while stronger hydrogen bonding established between drugs and the materials in basic conditions decreased the drug release rate.

Jiang et al. developed a pH-responsive dansyl-modified β-cyclodextrin [302]. It seems that the combination of hydrogen bonding and hydrophobic interactions allowed constructing pH-responsive self-assemblies with promising application potential for controlled drug release.

New CD derivatives developed by Mendonça et al. have proved efficiency in the delivery of antisense oligonucleotides to treat Huntington’s disease [303]. The non-cytotoxicity of materials in striatal neurons and Huntington’s fibroblast cells recommends them as a promising solution for neurodegenerative diseases.

Treatment with 2-hydroxypropyl-β-CD has an effect on autophagy, as well as on the proteins regulating dopaminergic neuronal differentiation in neuronal cultures and brain organoids from patients with Parkinson’s disease [304]. The dopaminergic differentiation was improved by modifying the levels of proteins involved in dopaminergic differentiation, autophagy, apoptosis, and neuroinflammation.

These examples illustrate the versatility of cyclodextrin inclusion complexes in enhancing drug solubility, stability, and delivery performance, laying the groundwork for their translation into marketed formulations, as discussed in the following section.

### 5.5. Market for Mulations and Clinical Applications

In spite of their controlled charge distribution, depending on the individual body and disease progress, there are numerous materials that include CDs on the drug market [305].

On the market, there are different formulations in which cyclodextrins have been added for different purposes (Table 7). Thus, a modified sulfobutylether-β-CD, known as Captisol^®^ is present in 13 FDA-approved injectables and other clinical formulations [158].

CAVAMAX^®^ W6 FOOD is a CDs-based product from Wacker Chemie AG that can be used to stabilize food foams and oil-in-water emulsions.

The toxicological profile of chemically modified cyclodextrins has been extensively studied, especially for derivatives used in clinical formulations such as hydroxypropyl-β-cyclodextrin (HP-β-CD) and sulfobutylether-β-cyclodextrin (SBE-β-CD). While native β-CD exhibits nephrotoxicity when administered parenterally due to renal accumulation [306,307,308], its modified analogs demonstrate improved safety. HP-β-CD, for instance, is approved for oral and intravenous use and is classified as generally recognized as safe (GRAS) by the FDA, though high doses can still cause renal vacuolization [309,310]. SBE-β-CD (Captisol^®^), used in multiple marketed injectables, has shown low toxicity in humans at therapeutic doses and is cleared primarily via the kidneys [119,311,312]. Toxicity is generally dose-dependent and route-specific; intravenous administration requires careful control of concentration and volume. Regulatory agencies have established maximum acceptable daily intakes (e.g., 16 mg/kg for HP-β-CD and 250 mg/kg for SBE-β-CD in parenteral formulations) [309,313,314]. Despite their favorable profiles, long-term and high-dose administration, particularly in populations with compromised renal function, remains a concern that warrants continued drug safety monitoring.

The regulatory status of cyclodextrins varies across pharmaceutical markets but has been increasingly clarified over recent decades. In the United States, the FDA has granted generally recognized as safe (GRAS) status to several cyclodextrins, including α-, β-, and γ-cyclodextrin for certain uses, and has approved derivatives such as hydroxypropyl-β-cyclodextrin (HP-β-CD) and sulfobutylether-β-cyclodextrin (SBE-β-CD, Captisol^®^) for parenteral administration in specific formulations [315]. The European Medicines Agency (EMA) has also approved multiple cyclodextrin-based excipients; for example, HP-β-CD is included in the European Pharmacopoeia, and its use is permitted in both oral and injectable drug products [24,66,113,316]. The EMA and FDA have both issued guidance regarding acceptable daily intake levels, safety margins, and renal function considerations, particularly for parenteral administration. In both regions, the use of chemically modified CDs is considered acceptable when supported by sufficient toxicological and pharmacokinetic data, and regulatory approvals are typically granted on a product-specific basis rather than for cyclodextrins as a general class [38,57,317,318].

Numerous CD-based formulations have been successfully commercialized, with their safety and effectiveness having been validated. HP-β-CD is an excipient in the intravenous formulation of itraconazole (Sporanox^®^ IV), which enhances its solubility and avoids the use of harmful co-solvents [238]. Similarly, SBE-β-CD is used in the formulation of intravenous voriconazole (Vfend^®^) and ziprasidone (Geodon^®^) [319].

Sugammadex (Bridion^®^), a modified γ-CD, is an FDA-approved drug used to reverse neuromuscular blockade induced by rocuronium and vecuronium during surgery. It works by encapsulating the neuromuscular blocking agents, forming tight complexes that are excreted renally. This drug is a striking example of a CD not just as a carrier but as an active pharmaceutical ingredient.

Several eye drop formulations use CDs to enhance drug solubility and stability. For example, dexamethasone/HP-β-CD-based formulations have shown superior bioavailability and less ocular irritation than conventional solutions. CD complexes have also improved the delivery of hydrophobic drugs in dermatological and nasal formulations.

In the field of gene delivery, CD-based vectors are being tested for plasmid and siRNA delivery. Their ability to compact nucleic acids and facilitate endosomal escape makes them attractive alternatives to viral vectors, especially considering their low immunogenicity [320].

CDs are also used in vaccine delivery systems. Some COVID-19 mRNA vaccine candidates incorporate CDs into lipid nanoparticles to improve stability and intracellular delivery. These multifunctional systems enhance immune responses while reducing cold-chain dependency.

Looking ahead, CDs are being integrated into 3D-printed dosage forms, smart patches, and implantable pumps, expanding their utility in personalized and digital health applications. Their regulatory acceptance, biocompatibility, and multifunctional properties ensure that CDs will continue to play a central role in pharmaceutical innovation.

Emerging trends also include CD-based inhalation therapies for respiratory diseases, oral delivery of peptide drugs, and targeted photodynamic therapy. The versatility of CDs, combined with ongoing advancements in materials science and biotechnology, continues to open new avenues for clinical applications and pharmaceutical breakthroughs.

## 6. Conclusions and Future Perspectives

Cyclodextrins (CDs) have evolved from enzymatic starch derivatives into multifunctional agents in supramolecular chemistry and pharmaceutical sciences. Their distinct architecture, with a hydrophilic exterior and hydrophobic cavity, enables them to form non-covalent inclusion complexes with a wide range of guest molecules, thereby improving solubility, stability, and bioavailability.

The structural variety among α-, β-, and γ-CDs, along with chemically modified derivatives (e.g., hydroxypropyl-, sulfobutyl ether-, methylated, and carboxymethylated CDs), expands their practical applications. These modifications address limitations such as poor aqueous solubility and low complexation specificity and introduce advanced functionalities like pH responsiveness and polymer compatibility.

Characterization techniques, including NMR, ITC, FTIR, XRD, and MS, together with computational tools like molecular dynamics and machine learning, support the rational design of CD-based complexes by elucidating binding affinities, stoichiometry, and thermodynamics.

Pharmaceutically, CDs play a critical role in solubilizing poorly water-soluble drugs, affecting nearly 40% of new chemical entities. Their inclusion complexes protect active ingredients from hydrolysis, oxidation, and photodegradation, extend shelf life, and enable optimized release profiles across various administration routes (oral, parenteral, ophthalmic, nasal, and transdermal).

Emerging CD-based delivery platforms (nanosponges, micelles, hydrogels, nanogels, and dendrimers) allow for controlled and site-specific drug release. Many of these systems are responsive to environmental stimuli such as pH, redox potential, enzymes, and temperature, particularly advantageous for oncology and chronic diseases.

Functionalized CDs conjugated with targeting ligands (e.g., folate, transferrin, peptides, antibodies) enhance site-specific delivery and are being explored for crossing the blood/brain barrier, opening new possibilities for treating central nervous system conditions. CDs are also promising tools in non-viral gene delivery due to their ability to encapsulate and protect nucleic acids.

Beyond pharmaceutical applications, CDs are employed in food technology, agriculture, environmental remediation, textiles, and diagnostics. Their capacity to encapsulate volatile compounds, neutralize odors, remove pollutants, and stabilize formulations highlights their versatility across industries.

Advanced CD systems, responsive to multiple stimuli, are in the leading position of smart material design. These multi-functional platforms offer precise control over drug delivery and therapeutic performance, allowing adaptation to complex biological environments.

Despite significant progress, challenges persist. CDs have limited drug-loading capacity, and some derivatives raise concerns about toxicity, regulatory approval, or manufacturing scale-up. A deeper understanding of host–guest specificity, in vivo behavior, and dissociation kinetics remains necessary.

Future efforts should integrate high-throughput screening, nanotechnology, and systems biology to create next-generation CD carriers. Interdisciplinary collaboration will be key to translating these materials into clinical and industrial applications.

In summary, CDs are highly tunable, biocompatible systems at the intersection of natural product chemistry and advanced material science. Their continued development promises significant impact on therapeutic delivery, diagnostics, and sustainable technologies across sectors.

## Figures and Tables

**Table 1:** Main areas of cyclodextrin use.

Application Area	Key Functions and Benefits	References
Pharmaceuticals	Enhance drug solubility, stability, bioavailability; mask taste/smell; enable targeted and controlled drug delivery (oral, ocular, nasal, parenteral, topical); used in nanomedicine and cancer therapy	[64,65,66,67,68,69]
Food industry	Improve flavor, aroma, and shelf life; stabilize sensitive nutrients; remove cholesterol; act as food additives; extend storage period	[65,70,71,72]
Cosmetics and toiletries	Stabilize fragrances and active ingredients; improve product texture and shelf life	[68,70,71]
Environmental and industrial	Remove pollutants (e.g., pesticides, heavy metals); wastewater treatment; oilfield chemicals; used in nanomaterials and coatings	[71,73,74,75]
Agriculture	Enhance pesticide formulation and remediation; protect and deliver agrochemicals	[71,76]
Advanced materials	Used in nanotechnology (nanoparticles, nanofibers, nanomicelles); biomaterials; tissue engineering	[67,73,74]

**Table 2:** Solubility enhancement by cyclodextrin type.

Cyclodextrin Type	Drug/Compound	Solubility Increase	References
HP-β-CD	Myricetin	31.45×	[96]
HP-β-CD	Kaempferol	12.7×	[103]
SBE-β-CD (low-melting mix)	Fluticasone propionate	4000×	[104]
SBE-β-CD	Pomalidomide	Highest among 9 CDs	[105]

**Table 3:** Reaction yields and physicochemical parameters in CD derivatization pathways.

Derivatization Pathway/System	Reaction Yield/Efficiency	Key Physicochemical Parameters	References
Cross-linking CD with epichlorohydrin (ECH)	Up to 67.1% (drug loading, α-CDNS)	Optimal: 6 h solubilization, ECH/CD molar ratio 8:1, NaOH 33%; affects drug loading and particle size	[152]
Mechanochemical synthesis of insoluble CD polymers	Not specified (focus on reproducibility)	CD/reagent ratio determines solubility; mechano-chemical methods yield insoluble polymers; high reproducibility	[153]
CD–metal organic frameworks (CD-MOFs) for enrichment and derivatization	82.4–87.5% (relative recovery)	α-, β-, γ-CD-MOFs; RSD < 10.9%; LOD 0.934–2.77 ng/g; cavity size affects encapsulation and derivatization	[154]
Regioselective radical C–H trifluoromethylation (with CD)	Good yield (exact % not specified)	CD increases regioselectivity by stabilizing intermediates; metal salts increase yield	[155]

**Table 4:** Key factors affecting stoichiometry.

Factor	Effect on Stoichiometry	Example	References
Molecular size and shape	Small guests often form 1:1 complexes; larger or elongated guests may form 1:2 or 2:1	Naphthalene forms 1:2 with α-CD, 2:1 and 2:2 with β-CD; vanillin forms 1:1 with β-CD	[202,227]
Type of cyclodextrin	α-, β-, and γ-cyclodextrins have different cavity sizes, affecting possible stoichiometry	Larger γ-CD can accommodate larger guests or multiple guests	[227,228,229]
Guest polarity and interactions	Hydrophobic and hydrogen bonding interactions stabilize complexes, influencing stoichiometry	Hydrophobic effects and electrostatic forces mediate 1:1 complexes with amino acids	[230,231,232]
Solvent environment	Solvent can alter complex stability and stoichiometry	Deep eutectic solvents can promote 2:1 complexes	[228,233]
Concentration	Higher concentrations can favor higher-order complexes (e.g., 1:2, 2:1)	5-fluorouracil forms both 1:1 and 1:2 complexes with β-CD depending on conditions	[111,234]

**Table 5:** Measured enhancements in drug formulation.

Drug/Complex	Cyclodextrin Type	Solubility Increase	Bioavailability Increase	Half-Life/ Other Stability	References
Oxymetholone	Carboxymethyl-β-cyclodextrin	~3-fold	Not specified	Improved stability	[243]
Progesterone	Sulfobutyl-ether-β-cyclodextrin	~7000-fold	5-fold (in rats)	Prevents precipitation	[118]
Ibuprofen	Porous carboxymethyl-β-cyclodextrin polymer	Not specified	~4-fold (AUC), 3-fold (Cmax)	Enhanced release	[253]
Lisinopril	β-cyclodextrin	Not specified	2-fold	Stable after 3 months	[254]
Flutamide	β-cyclodextrin	5.7-fold	Implied by solubility	Rapid release	[255]
Altrenogest	Hydroxypropyl-β-cyclodextrin	1026-fold	1.14-fold (relative)	Improved dissolution	[256]
Curcumin	β-cyclodextrin/nanosponge	2.3–2.95-fold	Not specified	Higher stability constant	[257]
Cyclolinopeptides	β-cyclodextrin (Pickering emulsion)	Not specified	13.6-fold	Higher physical stability	[258]
Dexamethasone (ocular)	Thiolated hydroxypropyl-β-cyclodextrin	Not specified	11.7-fold (AUC, ocular)	4-fold longer residence	[146]

**Table 6:** Selected cyclodextrin inclusion complexes: guest molecules, CD type, and observed effects.

Guest Molecule	Cyclodextrin Type	Observed Effect	References
Curcumin	Hydroxypropyl-β-CD	Improved solubility and nasal/olfactory tissue permeation	[241,284]
Albendazole	Methyl-β-CD	Solubility increased by 150,000-fold; enhanced antitumor efficacy	[248,285]
Ibrutinib	Hydroxypropyl-β-CD (cross-linked)	Controlled release; improved solubility and bioavailability	[66,245]
Abiraterone acetate	2,6-di-O-methyl-β-CD	Enhanced dissolution rate; potential in prostate cancer treatment	[286,287,288]
Oxymetholone	Carboxymethyl-β-CD	Improved encapsulation and prolonged release	[243]
Genistein	Amino-appended β-CD	1000-fold solubility increase; enhanced antioxidant activity	[289,290]
Acyclovir	Pluronic127 + α/β-CD	Sustained release for topical application	[246,291,292]
Insulin	Various β-CD derivatives	Enhanced solubility, stability, and mucosal absorption	[268,293,294,295]
Doxorubicin	Modified β-CD conjugates	Targeted cancer cell delivery; increased cytotoxicity	[296,297,298]
Fenofibric acid	β-CD and methyl-β-CD	Controlled release; enhanced pharmacological profile	[273,299,300]

**Table 7:** Marketed cyclodextrin-based pharmaceutical formulations.

Product Name	Active Ingredient	CD Type	Formulation	Application Field	Manufacturer
Sporanox^®^ IV	Itraconazole	HP-β-CD	Intravenous	Antifungal	Janssen Pharmaceuticals
Vfend^®^	Voriconazole	SBE-β-CD (Captisol^®^)	Intravenous	Antifungal	Pfizer
Geodon^®^	Ziprasidone	SBE-β-CD (Captisol^®^)	Intramuscular	Antipsychotic	Pfizer
Bridion^®^	Sugammadex	γ-CD derivative	Intravenous	Reversal of neuromuscular blockade	Merck & Co.
Ozurdex^®^	Dexamethasone	HP-β-CD	Ophthalmic implant	Macular edema	Allergan
Abilify^®^ Injection	Aripiprazole	HP-β-CD	Intramuscular	Schizophrenia, bipolar disorder	Otsuka Pharmaceutical
Cerenia^®^	Maropitant citrate	SBE-β-CD (Captisol^®^)	Injectable (veterinary)	Antiemetic (dogs, cats)	Zoetis

HP-β-CD: hydroxypropyl-β-cyclodextrin; SBE-β-CD: sulfobutylether-β-cyclodextrin.
